# Rumen DNA virome plasticity and viral metabolic potential are associated with seasonal adaptation in grazing yak and cattle on the Qinghai-Tibet Plateau

**DOI:** 10.1186/s40104-026-01487-8

**Published:** 2026-09-01

**Authors:** Wei Guo, Yinshu Yu, Weiwei Wang, Jiangkun Yu, Mi Zhou, Ruijun Long

**Affiliations:** 1 Key Laboratory of Plateau Mountain Animal Genetics, Breeding and Reproduction, Ministry of Education, College of Animal Science, Guizhou University, Guiyang, 550025 China; 2 Faculty of Land and Food Systems, The University of British Columbia, Vancouver, BC V6T 1Z4 Canada; 3 State Key Laboratory of Grassland and Agro-Ecosystems, International Centre for Tibetan Plateau Ecosystem Management, College of Ecology, Lanzhou University, Lanzhou, 730000 China; 4 State Key Laboratory for Conservation and Utilization of Bio-Resources in Yunnan, School of Life Sciences, Yunnan University, Kunming, Yunnan 650091 China

**Keywords:** Auxiliary metabolic genes, Cattle, Rumen DNA virome, Seasonal adaptation, Yak

## Abstract

**Background:**

As a diverse and abundant component of the rumen ecosystem, viruses interact with other microorganisms and are thought to influence microbial metabolism and host productivity. However, how the rumen virome responds to seasonal fluctuations in extreme environments remains poorly understood. Here, metagenomic analyses were used to investigate temporal dynamics of viral diversity, functional potential, and virus-host associations in the rumen virome of yak and cattle on the Qinghai-Tibet Plateau across warm and cold seasons.

**Results:**

Rumen viral communities exhibited pronounced seasonal variation in both yaks and cattle, with higher alpha diversity observed during the cold season than in the warm season. Across seasons, the yak rumen virome showed greater alpha diversity and community stability than that of cattle. In total, 27,353 temperate and 31,976 virulent viral operational taxonomic units (vOTUs) were identified, predominantly belonging to the class Caudoviricetes. These viruses were linked to microbial hosts spanning 24 bacterial and 8 archaeal phyla, with Bacteroidota and Bacillota representing the dominant lineages. Virus-host associations were more numerous in the cold season and showed distinct host-specific patterns between yaks and cattle. Cold-season virome exhibited reduced diversity of anti-defense genes and enrichment of auxiliary metabolic genes (AMGs) associated with fatty acid metabolism and hemicellulose degradation. Notably, greater divergence between yaks and cattle was observed during the cold season: the yak rumen virome was enriched in pathways related to amino acid, lipid, and energy metabolism, as well as cellulose-degrading CAZyme families, whereas the cattle rumen virome showed enrichment in general carbohydrate metabolism and replication and repair processes.

**Conclusion:**

Seasonal plasticity of rumen DNA virome and pronounced interspecific divergence between yaks and cattle provide insight into their distinct microbial processes in the harsh environment of the Qinghai-Tibet Plateau. These findings suggest that the rumen DNA virome exhibits complex ecological and functional responses to seasonal variation and may be associated with host-microbiome interactions and nutrient utilization under environmental stress. This study highlights the ecological relevance of rumen viral genomes in understanding virus-microbiome interactions, microbial adaptation, and nutrient utilization in high-altitude ruminants.

**Supplementary Information:**

The online version contains supplementary material available at https://doi.org/10.1186/s40104-026-01487-8.

## Introduction

The rumen harbors a vast and diverse community of microorganisms, including bacteria, archaea, fungi, protozoa, and viruses [1]. These microorganisms function synergistically to degrade and utilize recalcitrant plant materials (i.e., cellulose and hemicellulose), supplying a substantial portion of the energy and amino acids to meet the host’s requirements [2]. Consequently, growing efforts have been directed toward understanding how the rumen microbiome influences host health and economically important traits, including feed efficiency, methane emissions, and milk and meat production [3–9]. However, most of these efforts have centered primarily on rumen bacteria, archaea, protozoa, and fungi, leaving viruses largely overlooked despite their high abundance and modulatory effects on rumen microbial communities [10, 11].

Viruses represent a distinct and abundant component of the rumen ecosystem, with densities ranging from 5 × 10^7^ to 1.6 × 10^10^ particles per milliliter of rumen fluid [12]. Rumen viruses primarily exhibit lytic or temperate lifestyles, and some encode auxiliary metabolic genes (AMGs) that can modulate microbial host metabolism during infection [13, 14]. By targeting core microbial taxa involved in key metabolic pathways, viruses may further modulate the rumen fermentation process [15, 16]. Given these multifaceted interactions, the functional potential of rumen virome has attracted considerable attention. For instance, Yan et al. [11] cataloged 397,180 species-level viral operational taxonomic units (vOTUs), greatly expanding the known diversity of the rumen virome. Subsequent analyses revealed that many rumen viruses are associated with key microbial taxa, including fiber-degrading bacteria and methanogens [17]. Moreover, accumulating evidence suggests that the rumen virome is linked to host phenotypes, such as feed efficiency, lactation performance, weight gain, and methane emissions [11, 12, 18]. Notably, viruses predicted to infect *Methanobrevibacter* have also been associated with increased carcass weight in beef cattle [19]. Collectively, these findings indicate that rumen viruses interact closely with core microbial populations and may contribute to host phenotypic variation, underscoring their important role in shaping rumen ecosystem and animal productivity. Despite the growing body of knowledge regarding the rumen virome in beef and dairy cattle under controlled or captive conditions [12, 13, 18–20], its composition and functional potential in grazing ruminants reared under extreme environments remain poorly understood. Characterizing the composition, functional potential, and host interactions of the rumen virome in these populations may provide novel insights into virus-host ecological interactions and the adaptive roles of rumen viruses under seasonal environmental pressures.

The yak (*Bos grunniens*) is an emblematic herbivore species of the Qinghai-Tibet Plateau, characterized by physiological and metabolic adaptations that facilitate survival amidst hypoxia, thermal stress, and nutritional deficits [21]. Accumulating evidence indicates that these adaptive strategies are mediated, at least in part, by the rumen microbiome. Relative to low-altitude cattle (*Bos taurus*), yak maintains a distinct rumen microbial consortium that optimizes fiber degradation and fermentation efficiency, thereby improving feed utilization [22, 23]. Moreover, the rumen microbiome of grazing yak exhibits superior seasonal resilience and adaptive capacity, particularly during the cold season [24]. Considering the pivotal role of viruses in shaping microbial structure and metabolic function [13, 17], the rumen virome is posited as a potential driver of these seasonal microbial dynamics. However, most yak rumen virome studies have focused on captive populations [25, 26]. Since viral communities are profoundly driven by animal species, diet and environmental factors [13, 25], such studies may mask the unique viral dynamics of free-grazing ruminants, potentially contributing to host adaptation. Accordingly, we hypothesized that the yak rumen virome exhibits seasonal dynamics distinct from those of cattle and that these differences may be associated with host adaptation to the harsh environmental conditions of the Qinghai-Tibet Plateau [21]. To test this hypothesis, we performed a longitudinal metagenomic analysis to characterize the composition, functional potential, and virus-host interactions of the rumen DNA virome in grazing yaks and cattle. This study provides new insights into the ecological and functional roles of rumen viruses in high-altitude ruminants and advances our understanding of microbiome-mediated adaptation to seasonal nutritional and environmental stresses, with potential implications for improving feed utilization efficiency and resilience of grazing livestock in harsh environments.

## Materials and methods

### Animal experiment and sample collection

Metagenomic sequencing data used in the current study were obtained from our prior study [24]. The cohort consisted of 12 castrated males [6 cattle (*Bos taurus*: Qaidam cattle, body weight: 230 ± 15 kg) and 6 yaks (*Bos grunniens*: Tianzhu White yak, body weight: 180 ± 20 kg); Age: 36 ± 4 months] co-grazing year-round on natural pasture on the Qinghai-Tibet Plateau without supplementation. Rumen fluid was sampled longitudinally using a stomach tube before morning grazing at four time points, corresponding to the cold (November and January) and warm (late April and August) seasons.

### Metagenome sequencing

Total DNA was extracted from all 48 rumen fluid samples following the protocol described previously [27]. After quality and quantity assessment, DNA samples were used to construct the metagenomic libraries, which were further purified and sequenced on an Illumina HiSeq X Ten platform (2 × 150 bp paired end; Illumina, San Diego, CA, USA) at Majorbio BioPharm Technology Co., Ltd. (Shanghai, China). Quality control of sequencing data was conducted as described previously [24]. In brief, raw data were processed using Trimmomatic (v.0.38) [28] to trim adapter sequences and discard low-quality bases (q < 25) and short reads (< 50 nt). After that, Bowtie2 [29] was employed to remove host DNA contamination by aligning high-quality reads to either the bovine (UMD 3.1.1) or yak (v1.1) genome. Clean reads were then de novo assembled into contigs using MEGAHIT (version 1.1.1) [30], followed by taxonomic assignment using Kraken2 [31].

### vOTU identification, taxonomy, lifestyle, and host prediction

Contigs longer than 1.5 kb in each sample were subjected to VirSorter2 (v2.2.4) (--min-score 0.5) [32] and DeepVirFinder (v1.0) (score > 0.9 and *P* < 0.05) [33] for viral prediction. All putative viral contigs were pooled and processed with CheckV (v0.7.0) to estimate genome completeness and remove host contamination [34]. Contigs classified as low-quality, medium-quality, high-quality, or complete were used for downstream analyses. These contigs were then dereplicated at 95% identity and 85% coverage using CD-HIT (v4.8.1) [35] to generate vOTUs. vOTUs were taxonomically classified using the PhaGCN module within PhaBOX2 (v2.1.12). Putative microbial hosts were predicted using iPHoP (v. 1.3.3) with a score threshold of 90 against the default database (iPHoP_db_Aug23_rw) [36], and viral lifestyle (virulent or temperate) was inferred using PhaTYP in PhaBOX2 with a score threshold of 0.5. To estimate vOTU abundance, clean reads from each sample were mapped against the non-redundant viral genomes using Bowtie2. SAM files were converted to sorted BAM format using SAMtools, and the abundance was quantified using CoverM (v 0.7.0) in contig mode [37]. Finally, the relative abundance of viral families or higher taxonomic levels was calculated as the proportion of viral genomes assigned to a specific taxon relative to the total number of annotated viral genomes.

### Functional annotation of viral genes

Open reading frames (ORFs) within each vOTU were predicted by Prodigal (v2.6.3) using the “-p meta” option, followed by dereplicating using MMseqs2 (v14-7e284) as previously described [38]. The resulting non-redundant gene set was functionally annotated against the KEGG database via KofamScan (v1.3.0), retaining hits with scores exceeding HMM-specific thresholds [39]. Carbohydrate-active enzymes (CAZymes) were identified by aligning the gene set against the CAZy database using DIAMOND with the parameters -e 1e-5 --min-score 60 --id 30. Viral anti-defense genes were identified from the same gene set using AntiDefenseFinder within the DefenseFinder framework (v2.0.2) in anti-defense specific mode (“-A”) [40]. The abundances of annotated functional genes (KOs, CAZymes, and anti-defense genes) were estimated using CoverM, following the same procedure used for vOTU abundance estimation.

### Statistical analysis

All statistical analyses were conducted using R software (v 4.5.0) or GraphPad Prism (v 8.0.1). For alpha-diversity analyses, we used the effective Shannon diversity, calculated as the exponential of the Shannon index at the vOTU level or functional gene level, and differences between seasons within each species and between host species within each season were assessed using the Wilcoxon rank-sum test, with *P* < 0.05 considered statistically significant. For beta diversity, principal coordinate analysis (PCoA) based on Bray–Curtis distances was conducted to characterize viral community composition (vOTU level) and functional profiles; dissimilarities were tested using PERMANOVA analysis via the *adonis* () function in the *vegan* R package. Differences in the relative abundance of temperate or virulent viruses were assessed using the Wilcoxon rank-sum test for comparisons between ruminant species within the same season and between seasons within the same species, with *P* < 0.05 considered statistically significant. Differential abundance analyses of KEGG pathways, CAZymes, anti-defense genes, viral taxa (vOTU and family levels), and microbial hosts (genus level) were performed using DESeq2 to compare cold versus warm seasons within each species and cattle versus yak within each season. Significance was defined as an adjusted *P* < 0.05. Finally, the correlations between the relative abundances of vOTUs and their microbial hosts were assessed using Spearman correlation analysis, with significance defined as *P* < 0.05.

## Results

### Overview of rumen virome

**Fig. 1:**
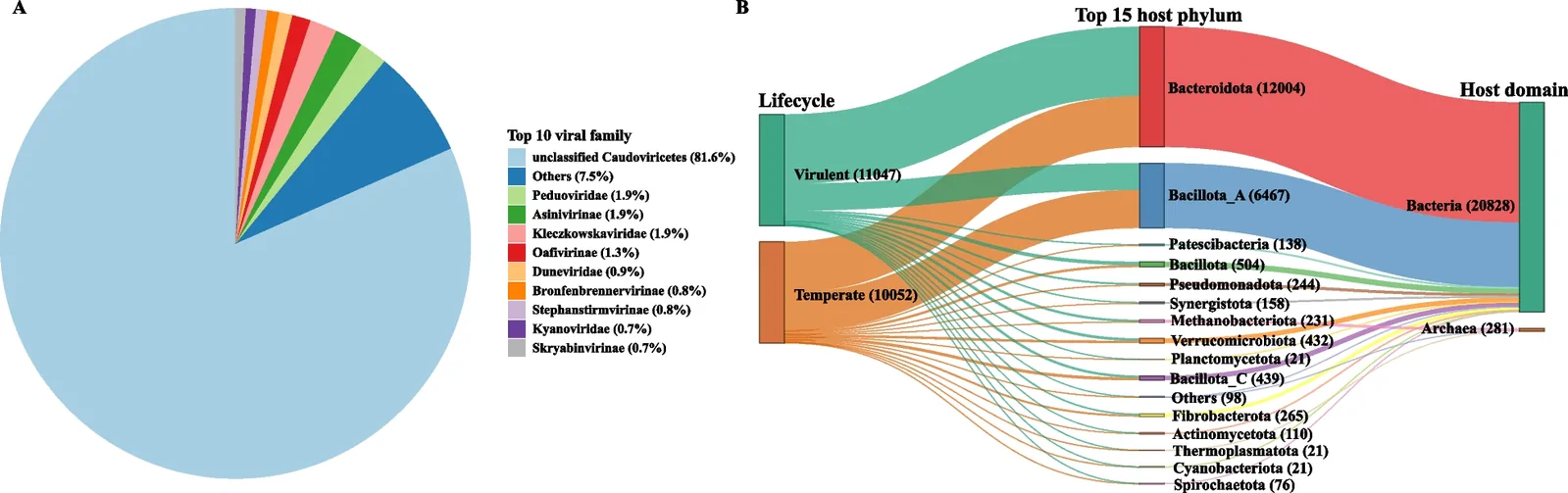
Viral community structure and predicted virus-host associations. **A** Top ten viral families based on mean relative abundance across all samples; remaining families are grouped as “Others”. **B** Predicted virus-host linkages. Numbers in parentheses indicate vOTU counts for each phage lifecycle type, host microbial phylum, and domain

Following quality control and dereplication (length > 1.5 kb), a total of 112,777 vOTUs were identified. Based on genome quality assessment, these were classified as complete (424), high-quality (508), medium-quality (888), and low-quality (110,957). Taxonomic analysis revealed that 57.35% (64,681) of the vOTUs could be successfully assigned to specific viral taxa (Additional file 1: Table S1). Among the annotated vOTUs, unclassified Caudoviricetes accounted for 81.6%, followed by Peduoviridae (1.9%), Kleczkowskaviridae (1.9%), Oafivirinae (1.3%), and Duneviridae (0.9%) (Fig. 1A). Lifestyle prediction identified 27,353 temperate and 31,976 virulent vOTUs (Additional file 1: Table S2). Approximately 48.8% (*n* = 48,308) of vOTUs were successfully assigned to microbial hosts spanning 8 archaeal and 24 bacterial phyla, with Bacteroidota being predominant (Additional file 1: Table S3).

By integrating taxonomic annotations with lifestyle and host predictions, 21,099 viruses were retained for downstream analysis. This subset included 11,047 virulent and 10,052 temperate viruses, the majority of which were predicted to infect the phylum Bacteroidota (*n* = 12,004; Fig. 1B). In total, 88,599 virus-host linkages were predicted (88,046 bacterial; 553 archaeal), comprising 51,640 virulent and 36,959 temperate vOTU-host linkages, respectively (Additional file 1: Tables S3 and S4). Notably, lifestyle-specific host associations were observed: Bacillota_B (bacteria) and Aenigmatarchaeota (archaea) were linked exclusively to temperate viruses (Additional file 1: Tables S3 and S4), whereas Methanobacteriota_B and Thermoproteota (archaea) were targeted solely by virulent viruses (Fig. 1B, Additional file 1: Tables S3 and S4). Host range analysis revealed that 14,794 vOTUs (70.1%) were predicted to infect multiple prokaryotic host genomes—primarily within the same genus (e.g., *Prevotella*)—whereas 6,305 vOTUs (29.9%) were restricted to a single host genome (Additional file 1: Tables S3 and S4). Among these microbial hosts, *Prevotella* (*n* = 5,414) was the most frequently predicted bacterial host, followed by *Cryptobacteroides* (*n* = 1,324), *Limimorpha* (*n* = 980), *Butyrivibrio* (*n* = 795), UBA4372 (*n* = 762), *Limivicinus* (*n* = 656), and *Ruminococcus* (*n* = 429). The predominant archaeal hosts were *Methanobrevibacter* (*n* = 138) and *Methanobrevibacter_A* (*n* = 115) (Additional file 1: Tables S3 and S4).

### Variation in rumen viral communities across seasons and host species

**Fig. 2:**
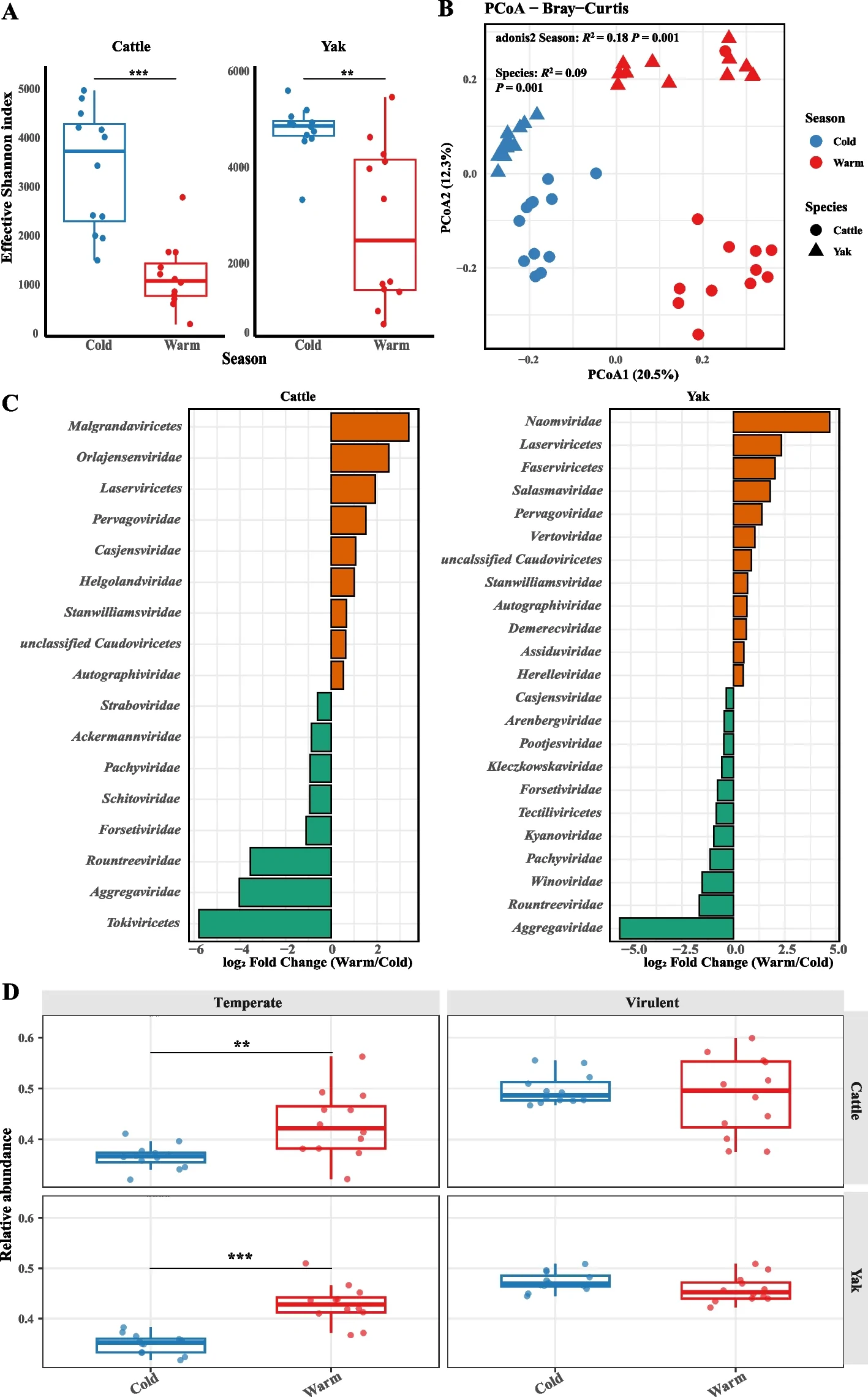
Seasonal variation in rumen viral community structure in yak and cattle. **A** Alpha diversity (Effective Shannon index) of viral communities. **B** Principal coordinates analysis (PCoA) of viral communities based on the Bray-Curtis distances. **C** Differentially abundant viral families between cold and warm seasons. **D** Relative abundance of virulent and temperate viruses across seasons. ^**^P < 0.01, ^***^P < 0.001

The Effective Shannon index was significantly higher in the cold season than in the warm season for both yaks and cattle (Fig. 2A, *P* < 0.05). Principal coordinates analysis (PCoA) revealed distinct clustering of the viral community driven by season (*R*^2^ = 0.18, *P* = 0.001) and species (*R*^2^ = 0.09, *P* = 0.001) (Fig. 2B). Notably, yaks consistently exhibited greater viral diversity than cattle across both the cold and warm seasons (Additional file 2: Fig. S1A, *P* < 0.05). Furthermore, yaks displayed lower compositional variation (Bray–Curtis distance) compared to cattle regardless of the season (Additional file 2: Fig. S1B, *P* < 0.05).

Differential abundance analyses were performed to identify viral families enriched or depleted across seasons and species. In total, 23 and 17 viral families were identified as differentially abundant between the cold and warm seasons in yaks and cattle, respectively (Fig. 2C). Furthermore, the relative abundance of temperate viruses was significantly higher in the cold season than in the warm season for both ruminant species (Fig. 2D, *P* < 0.05), whereas no such trend was observed for virulent viruses (Fig. 2D). When comparing the two host species, 19 viral families showed differential abundance between yaks and cattle in the cold season, with 9 families enriched in yaks and 10 enriched in cattle (Additional file 2: Fig. S2). In contrast, only 8 viral families differed between the two ruminant species in the warm season, including 4 families enriched in yaks and 4 enriched in cattle (Additional file 2: Fig. S2).

### Seasonal and host-associated variation in virus-encoded metabolic genes and CAZymes

Consistent with the taxonomic variation, the metabolic functional profiles of the rumen virome were significantly influenced by both season and host species (Additional file 2: Fig. S3). In total, 49,043 non-redundant ORFs were assigned to KEGG Orthologs (KOs) (Additional file 1: Table S5), and 30,455 were annotated as CAZymes (Additional file 1: Table S6). The top 10 viruses encoding the highest number of KOs were exclusively affiliated with the family Peduoviridae and were predicted to infect a diverse range of hosts, including *Bact-11* (1), *Prevotella* (3), *Saccharofermentans* (1), *Limimorpha* (2), *RF16* (1), *F23-D06* (1), and *Cryptobacteroides* (1) (Additional file 2: Fig. S4A). In contrast, viruses encoding the highest numbers of CAZyme genes (top 10) were all classified within the unclassified Caudoviricetes. These viruses were predicted to infect both fiber-degrading bacteria and methanogenic archaea, including *Prevotella* (4), *Butyrivibrio* (2), *Sodaliphilus* (2), *Methanobrevibacter_A* (1), and *Fibrobacter* (1) (Additional file 2: Fig. S4B).

**Fig. 3:**
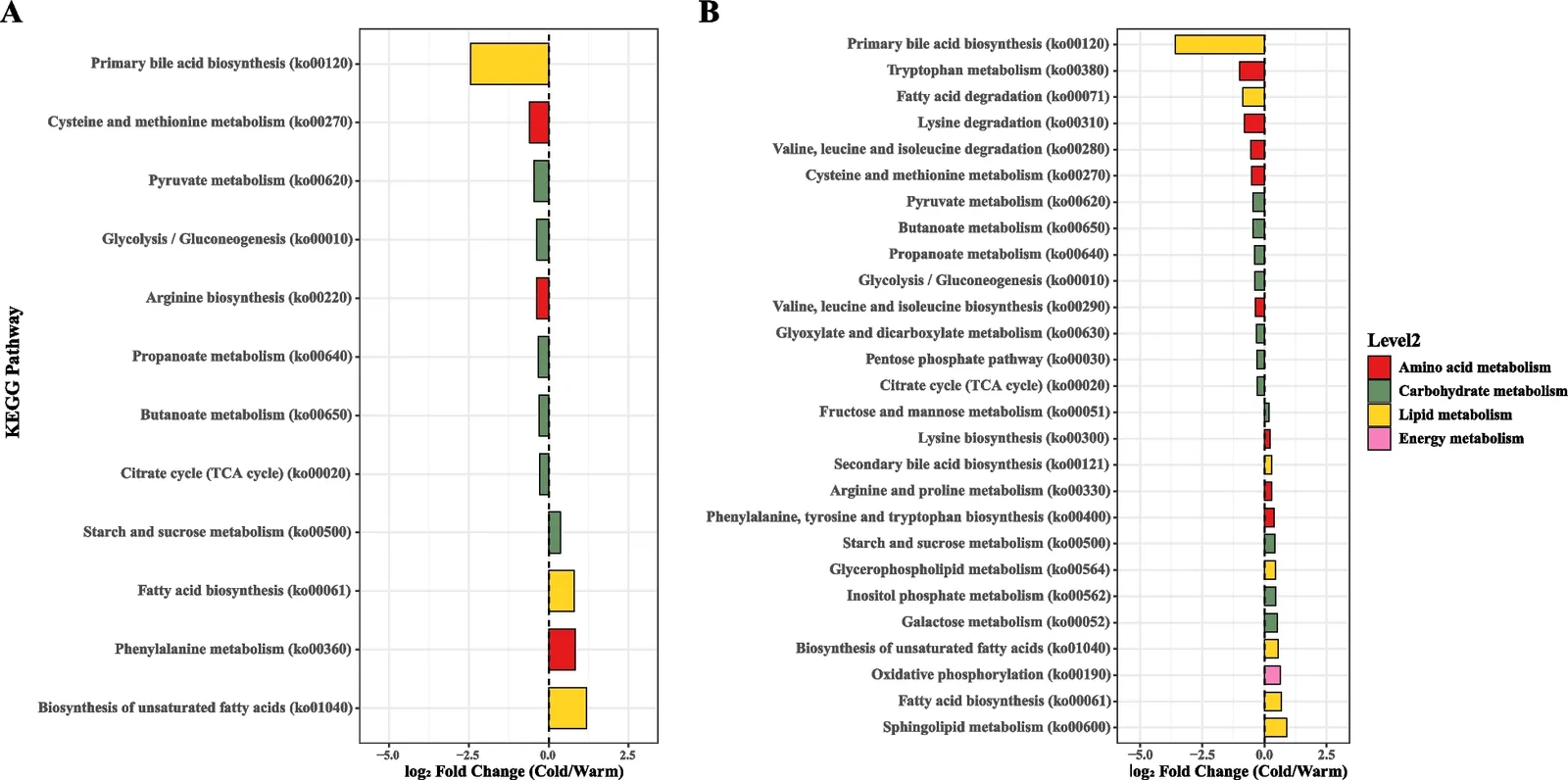
KEGG functional profiles of the rumen virome. Differentially abundant KEGG pathways between the cold and warm seasons in cattle (**A**) and yak (**B**)

**Fig. 4:**
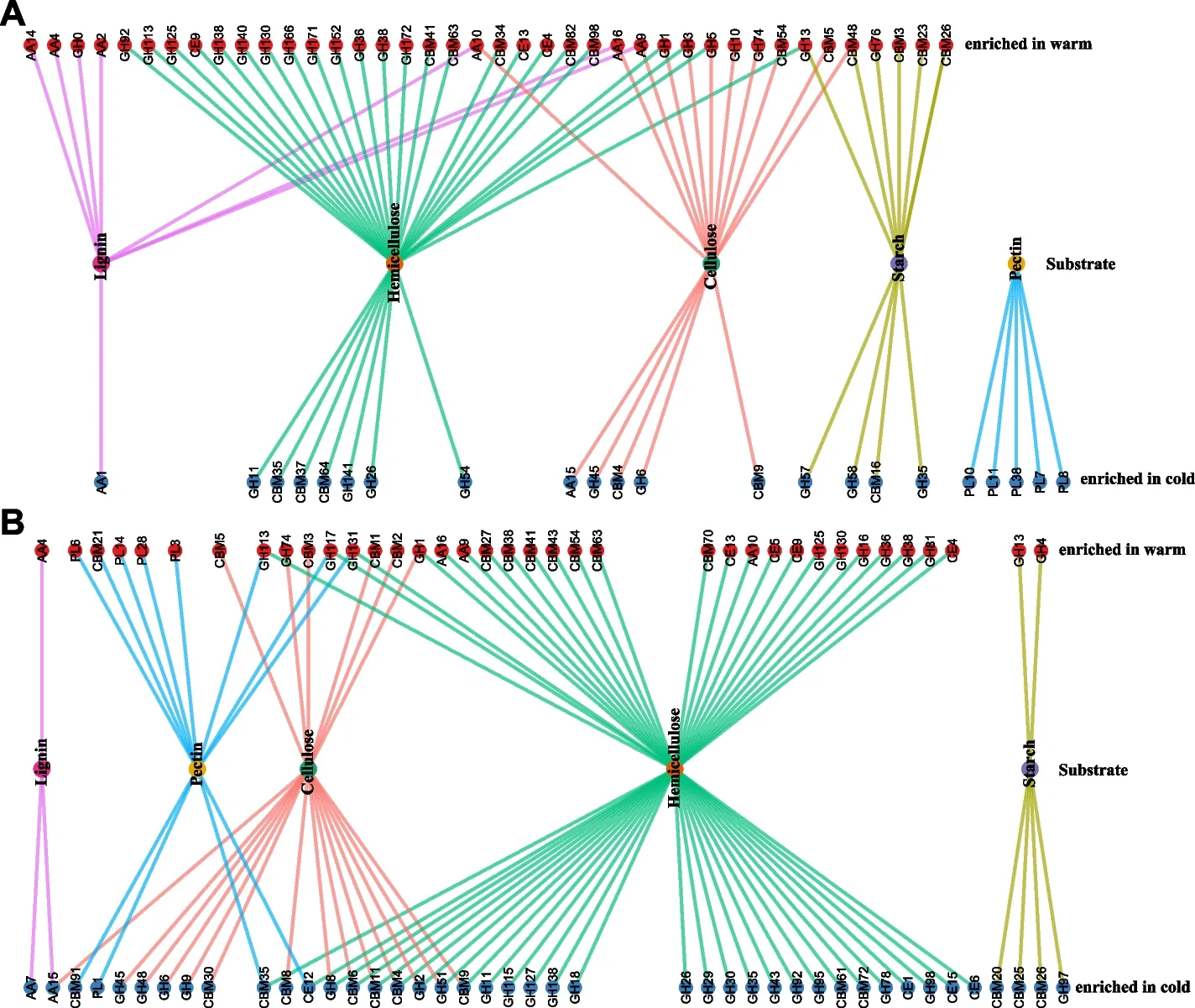
CAZyme profiles of the rumen virome. Differentially abundant CAZymes between the cold and warm seasons in cattle (**A**) and yak (**B**)

Seasonal shifts in community-level metabolic potential revealed 34 differentially abundant pathways in cattle, evenly distributed between the cold and warm seasons (17 each) (Additional file 1: Table S7). Pathways enriched in the warm season were primarily related to amino acid metabolism (cysteine and methionine metabolism and arginine biosynthesis) and carbohydrate metabolism (pyruvate metabolism, glycolysis/gluconeogenesis, propanoate metabolism, butanoate metabolism, and citrate cycle) (Fig. 3A). In contrast, pathways involved in fatty acid metabolism were enriched in the cold season (Fig. 3A). In yaks, 51 pathways showed significant differential abundance between seasons, with 26 enriched in the cold season and 25 in the warm season (Additional file 1: Table S7). Warm-season enrichment was dominated by amino acid metabolism (valine, leucine and isoleucine biosynthesis, cysteine and methionine metabolism, valine, leucine and isoleucine degradation, lysine degradation, and tryptophan metabolism) and carbohydrate metabolism (citrate cycle, pentose phosphate pathway, glyoxylate and dicarboxylate metabolism, glycolysis/gluconeogenesis, propanoate metabolism, butanoate metabolism, and pyruvate metabolism) (Additional file 1: Table S7; Fig. 3B). Conversely, the cold season was characterized by the enrichment of amino acid metabolism (phenylalanine, tyrosine and tryptophan biosynthesis, arginine and proline metabolism, and lysine biosynthesis), carbohydrate metabolism (galactose metabolism, inositol phosphate metabolism, starch and sucrose metabolism, and fructose and mannose metabolism), and lipid metabolism (sphingolipid metabolism, fatty acid biosynthesis, biosynthesis of unsaturated fatty acids, glycerophospholipid metabolism, and secondary bile acid biosynthesis) (Additional file 1: Table S7; Fig. 3B). For CAZymes, in cattle, 62 CAZyme families showed seasonal differences (22 cold-enriched; 40 warm-enriched), with those enriched in the warm season primarily targeting hemicellulose and cellulose degradation (Fig. 4A). In yaks, 79 CAZyme families showed differential abundance between seasons, including 42 enriched in the cold season and 37 enriched in the warm season, with the majority of cold-season enriched families associated with hemicellulose and cellulose degradation (Fig. 4B).

To further elucidate host-specific traits, we compared yaks and cattle across both seasons. Specifically, during the cold season, 40 KEGG pathways differed significantly between the two species, with 21 enriched in yaks and 19 in cattle (Additional file 2: Fig. S5, *P* < 0.05). Pathways overrepresented in yaks were predominantly related to amino acid metabolism, lipid metabolism, and energy metabolism, whereas those enriched in cattle were mainly involved in carbohydrate metabolism and replication and repair process (Additional file 2: Fig. S5, *P* < 0.05). During the warm season, pathways related to amino acid metabolism and lipid metabolism were consistently more abundant in yaks (Additional file 2: Fig. S5), while cattle showed enrichment in carbohydrate metabolism and metabolism of cofactors and vitamins (Additional file 2: Fig. S5, *P* < 0.05). Regarding CAZyme profiles, families involved in cellulose and hemicellulose degradation were significantly more abundant in yaks during the clod season, whereas these families were enriched in cattle during the warm season (Additional file 1: Table S8).

**Fig. 5:**
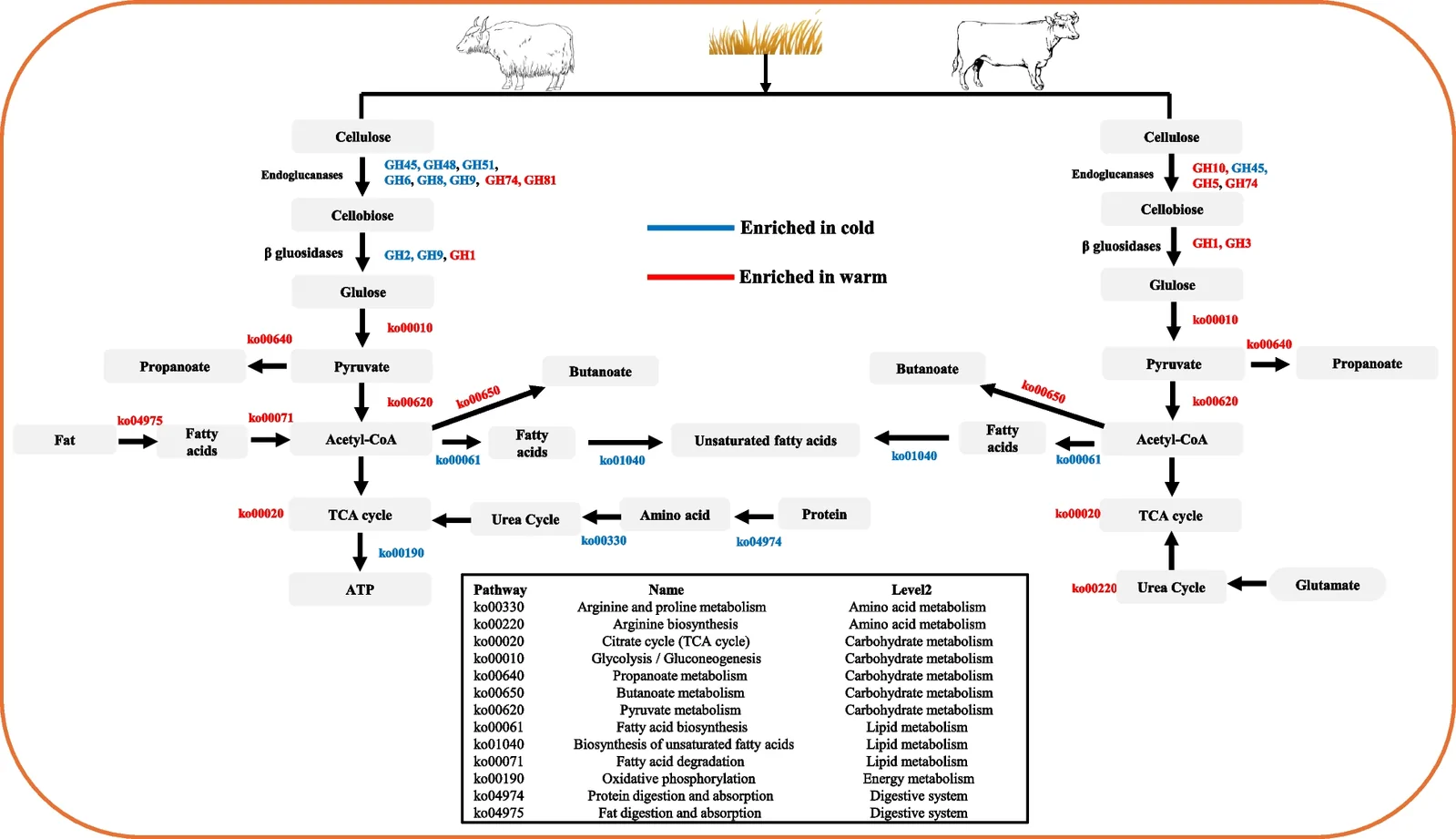
Integrative overview of viral metabolic potential in the rumen of yak and cattle, highlighting viral auxiliary metabolic genes (AMGs) and carbohydrate-active enzyme (CAZyme)-related functions

Finally, we integrated the differential KEGG pathways and CAZyme profiles of the rumen virome into a schematic to illustrate their potential roles in host adaptation across seasons (Fig. 5). This integrative model revealed that the yak rumen virome was enriched in amino acid, lipid, and energy metabolism, as well as CAZyme families involved in cellulose degradation (Fig. 5). In contrast, the cattle rumen virome was enriched in carbohydrate metabolism and replication and repair process (Fig. 5, Additional file 2: Fig. S5). During the warm season, although interspecies differences were reduced; yaks still exhibited higher representation of lipid metabolism (fat digestion and absorption and fatty acid degradation), whereas cattle remained enriched in carbohydrate metabolism (Fig. 5).

### Seasonal and host-associated patterns of viral anti-defense genes

**Fig. 6:**
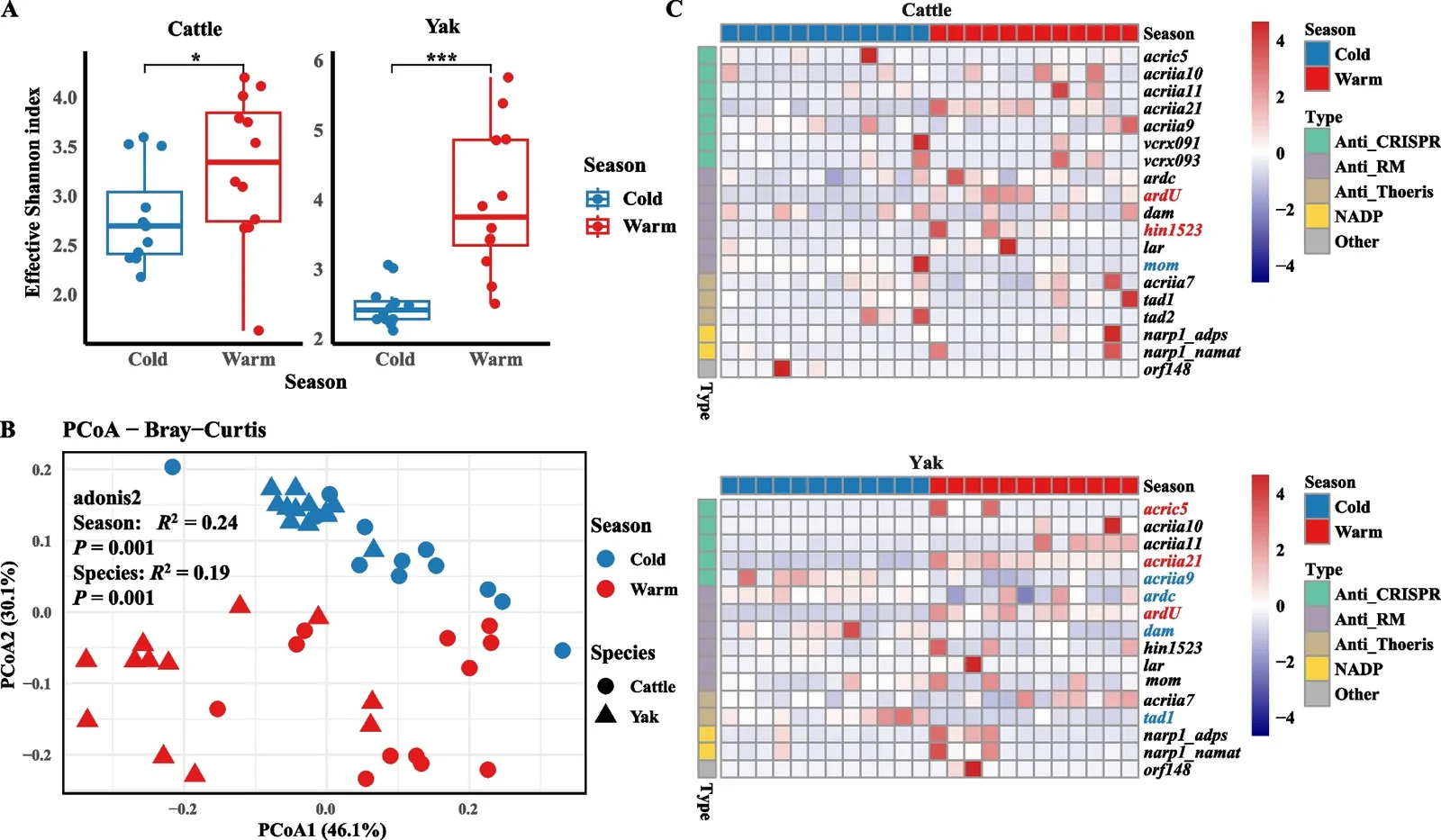
Seasonal dynamics of rumen virome-encoded anti-defense genes in yak and cattle. **A** Alpha diversity (Effective Shannon index) of anti-defense genes. **B** Principal coordinates analysis (PCoA) based on the Bray-Curtis distances. **C** Heatmaps showing the distribution of anti-defense genes across seasons; blue and red indicate enrichment in the cold and warm seasons, respectively. ^*^P < 0.05, ^***^P< 0.001

The rumen virome encodes a diverse array of anti-defense systems, including anti_CRISPR, anti_RM, anti_Thoeris, and NADP-associated genes (Fig. 6). The alpha diversity (Effective Shannon index) of anti-defense genes was significantly higher in the warm season than in the cold season in both yaks and cattle (Fig. 6A, *P* < 0.05). Principal coordinate analysis (PCoA) revealed distinct clustering patterns primarily driven by season, with host species exerting a weaker effect (Fig. 6B, *P* < 0.05). In cattle, three anti-RM genes (*ardU*, *hin1523*, and *mom*) displayed significant seasonal variation (Fig. 6C, *P* < 0.05). In yaks, a broader seasonal response was observed, with seven anti-defense genes displaying significant differences across seasons (Fig. 6C, *P* < 0.05). Overall, yaks and cattle harbored comparable numbers of anti-defense genes (16 and 19, respectively), although three genes were unique to cattle (Additional file 2: Fig. S6A). Gene-level comparisons further revealed species- and season-specific distributions of anti-defense genes (Additional file 2: Fig. S6B).

### Seasonal and host-specific effects on virus-host interactions in the rumen

**Fig. 7:**
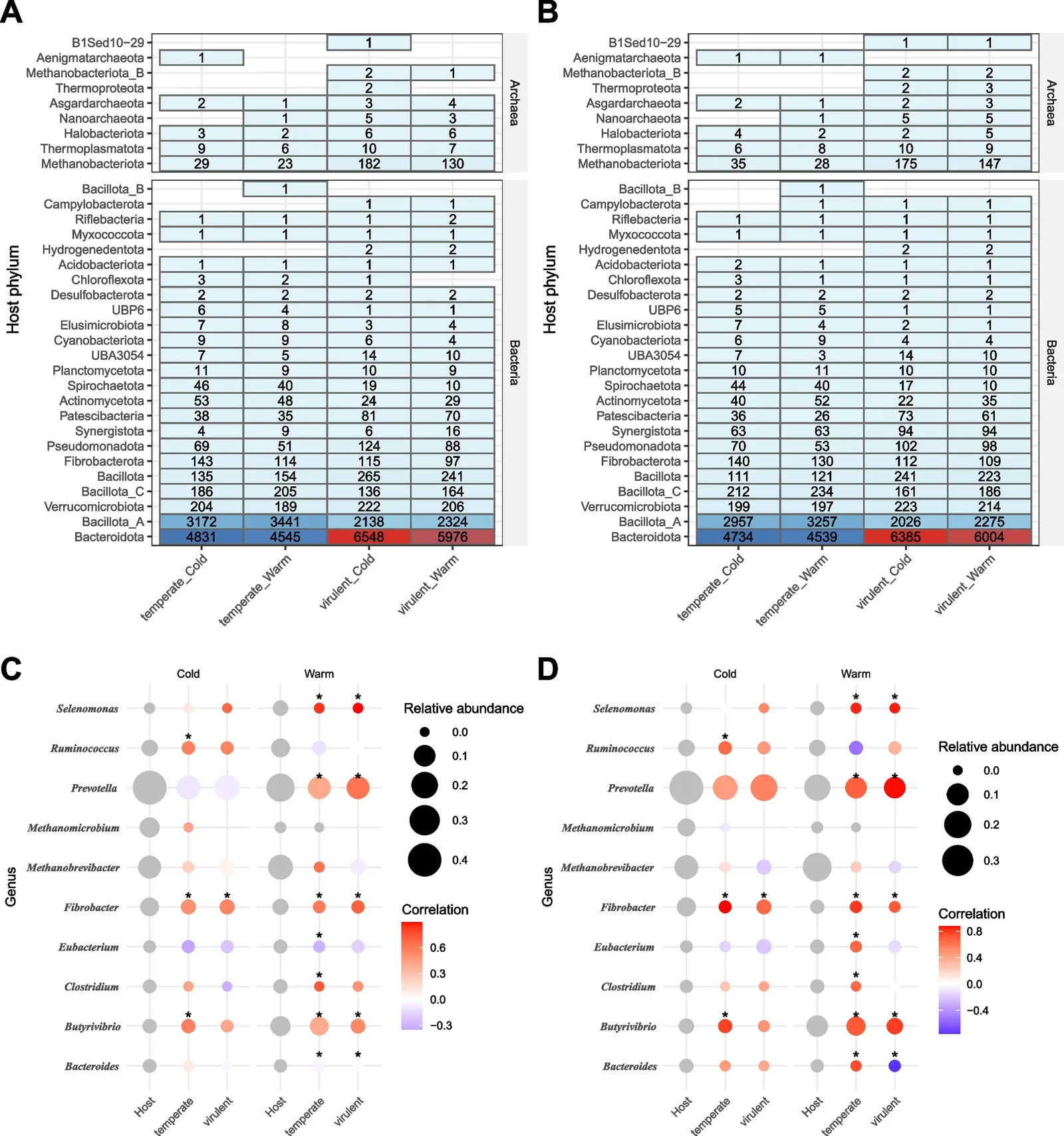
Seasonal patterns of virus-host interactions. Heatmaps showing vOTU distributions across host phyla in cattle (**A**) and yak (**B**). Numbers indicate vOTU counts. Spearman correlation analysis between viral and predicted host abundances in cattle (**C**) and yak (**D**). ^*^P < 0.05

By investigating predicted virus-host linkages, we found that both season and host species shape virus-host interaction patterns (Fig. 7A and B). A slightly higher number of predicted virus-host associations was observed during the cold season in both yaks (cold vs. warm: 79,835 vs. 78,481) and cattle (80,818 vs. 78,876) (Additional file 1: Table S9). Virulent viruses accounted for a greater proportion of these associations and exhibited a broader host range across bacterial phyla (31 phyla) compared with temperate viruses (yak: 29; cattle: 28) (Fig. 7A and B). Interestingly, a unique linkage between a temperate virus and Campylobacterota was unique to yaks during the warm season (Fig. 7B). At the genus level, *Prevotella* represented the most frequently predicted host in both yaks (cold = 5,133; warm = 4,841) and cattle (cold = 5,256; warm = 4,911), followed by *Cryptobacteroides* and *Limimorpha* (Additional file 1: Table S9). Several dominant bacterial genera, including *Butyrivibrio*, *Ruminococcus*, *Fibrobacter*, and *Methanobrevibacter*, showed increased virus-host associations in the cold season, whereas *Bacteroides* and *Eubacterium* exhibited host-specific seasonal patterns (Additional file 1: Table S9).

Correlation analysis of vOTU abundance and predicted microbial hosts further indicated that virus-host interaction dynamics were jointly shaped by both season and host species (Fig. 7C and D). In cattle, *Prevotella-*virus correlations shifted from negative in the cold season to positive in the warm season (Fig. 7C), whereas no consistent seasonal pattern was observed in yaks (Fig. 7D). Furthermore, virus-lifestyle-specific associations were detected in yaks during the warm season, in which *Bacteroides* abundance was negatively associated with virulent viruses but positively associated with temperate viruses (Fig. 7D). In the same season, *Methanobrevibacter* showed consistent virus-host associations across both cattle and yaks, being positively correlated with temperate viruses and negatively correlated with virulent viruses (Fig. 7C and D). Notably, *Fibrobacter*, *Butyrivibrio*, and *Selenomonas* exhibited robust and consistently positive correlations with viral abundance across seasons and host species (Fig. 7C and D).

## Discussion

As the “dark matter” of the rumen ecosystem, the DNA virome has attracted growing interest due to its important role in shaping microbial communities [17] and influencing host phenotypic traits [41]. Here, we comprehensively investigated the rumen DNA virome of grazing yaks and cattle by analyzing 48 metagenomes spanning cold and warm seasons on the Qinghai-Tibet Plateau. Our results revealed pronounced host- and season-dependent variation in viral community composition and functional potential, highlighting the remarkable plasticity of the rumen DNA virome under changing environmental conditions. Notably, viral divergence between the two species was more evident during the cold season, suggesting that environmental stress may strengthen host-specific ecological processes shaping viral communities. Collectively, these findings expand current understanding of rumen DNA viral ecology in high-altitude grazing ruminants and highlight the potential importance of viruses as dynamic regulators of microbiome responses to seasonal environmental fluctuations. Given the extreme environmental conditions of the Qinghai-Tibet Plateau, deciphering virome dynamics and virus-host interactions provides a valuable framework for understanding the microbial mechanisms underlying environmental adaptation and ecosystem resilience in grazing livestock.

Rumen DNA virome communities differed significantly across seasons and host species in the present study, consistent with previous observations [11, 13, 16]. These patterns suggest that seasonal environmental variation and host-associated factors jointly contribute to shaping viral community structure in the rumen. Notably, yaks consistently exhibited significantly lower Bray-Curtis distances than cattle in both warm and cold seasons, suggesting greater homogeneity in their rumen viral communities. Although seasonal variation in virulent viruses was not statistically significant, distinct trajectories were observed between yaks and cattle, with virulent phages increasing in yaks but decreasing in cattle during the cold season. The cold-season enrichment of virulent phages in yaks may indicate intensified virus-host interactions and enhanced lysis of microbial cells, which could contribute to the recycling of microbial biomass and nutrients within the rumen ecosystem under conditions of forage scarcity [42].

Consistent with recent studies [11, 19, 41], viruses belonging to the class Caudoviricetes dominated the rumen virome, suggesting that this group represents the major component of the currently characterized double-stranded DNA viruses in the rumen ecosystem. At the family level, Peduoviridae was the most abundant taxon in this study, whereas Siphoviridae has been reported as the predominant family in several previous rumen virome studies [12, 18, 25]. The observed differences likely reflect variation in host, environmental, and methodological factors among studies. The majority of predicted viral microbial hosts were assigned to the phyla Bacteroidota and Bacillota, and *Prevotella* was identified as the predominant genus, mirroring the host profiles reported in previous studies [19, 25, 41]. Similarly, the predicted archaeal viral hosts were predominantly affiliated with Methanobacteriota, particularly the genus *Methanobrevibacter*, consistent with previous reports [19, 25]. Together, these findings suggest that dominant bacterial and archaeal lineages may represent important hosts for rumen DNA viruses, highlighting their potential role in affecting virus-host interaction patterns within the rumen ecosystem. Furthermore, most viruses predicted to infect *Methanobrevibacter* were classified as virulent, consistent with previous reports indicating a predominance of lytic phages associated with this methanogenic genus in the rumen [18, 25, 43]. This pattern suggests that lytic virus-host interactions may represent an important component of archaeal viral ecology within the rumen ecosystem. Compared with temperate phages, lytic phages are generally more amenable to isolation and experimental characterization [44], making them attractive targets for future investigation. Moreover, recent studies have reported associations between methanogen-targeting phages and methane emissions [12, 41, 45], suggesting that viral predation may influence methanogen population dynamics. Further isolation and experimental validation of these viruses, including their host range and lytic activity, are needed to clarify their potential roles in regulating methanogenic communities and methane production in ruminants.

In the current study, most rumen DNA viruses were predicted to possess broad host ranges, consistent with recent reports [17, 46], highlighting the complex ecological interactions between viruses and microbial communities in the rumen ecosystem. Notably, the number of predicted virus-host linkages increased from the warm to the cold season in both yaks and cattle, suggesting intensified viral-microbial interactions under seasonal environmental stress. This pattern may reflect ecological restructuring of the rumen microbiome in response to reduced forage quality and nutrient availability during winter. The majority of virus-host associations involved dominant rumen taxa associated with fiber degradation, fermentation, and methanogenesis, including *Butyrivibrio*, *Ruminococcus*, *Fibrobacter*, and *Methanobrevibacter*, which have also been identified as major viral hosts in previous studies [17, 25]. These findings suggest that viruses may actively participate in regulating key microbial populations involved in energy acquisition and ecosystem functioning during periods of environmental challenge.

Beyond these general patterns, *Bacteroides* and *Eubacterium* exhibited contrasting seasonal interaction dynamics between the two host species. Specifically, cattle showed an increase in associations with virulent viruses during the cold season, whereas yaks exhibited a decline. As virulent phages are capable of influencing microbial community through host lysis [47], these contrasting patterns suggest that virus-host interactions involving these bacterial genera may respond differently to seasonal environmental conditions in the two host species. Although the functional implications of these patterns remain unclear, they suggest potential host-specific differences in virus-microbe interaction networks under seasonal environmental conditions.

Correlation analyses suggested the coexistence of lytic and lysogenic viral regulatory strategies within the rumen ecosystem. Negative associations between virulent viruses and hosts such as *Eubacterium* were consistent with density-dependent viral predation, as proposed by the kill-the-winner model [48]. In contrast, positive associations involving temperate phages and key rumen taxa, including *Methanobrevibacter*, *Fibrobacter*, *Clostridium*, and *Butyrivibrio*, were consistent with the piggyback-the-winner framework [49]. Notably, correlations between temperate viruses and the cellulolytic genus *Ruminococcus* shifted from negative in the warm season to positive in the cold season across both host species, indicating that viral regulatory modes may be dynamically adjusted in response to seasonal nutritional conditions. In addition, divergent correlations for *Prevotella* between yaks and cattle during the cold season may reflect host-specific microbial responses to winter environmental conditions. Together, these findings suggest that viruses may be important components of rumen microbial interaction networks, potentially influencing community dynamics under seasonal environmental variation.

Virus-carried AMGs can modulate host metabolism during infection and contribute to microbial adaptation under changing environmental conditions [50–55]. Consistent with previous rumen DNA virome research [18, 19], we identified a broad repertoire of AMGs involved in carbohydrate, lipid, and amino acid metabolism, highlighting the potential role of viruses in shaping microbial metabolic functions. Notably, AMGs associated with fatty acid metabolism and plant fiber degradation (e.g., GH45 and GH6) were enriched during the cold season and were primarily linked to *Prevotella* and *Butyrivibrio*. These functions may facilitate the utilization of recalcitrant winter forage and improve energy acquisition under nutrient-limited conditions [56, 57], which could in turn support microbial adaptation and help maintain ecosystem stability during seasonal environmental fluctuations [58]. Furthermore, AMG profiles differed between host species, particularly during the cold season, with genes involved in energy metabolism and lignocellulose degradation being significantly enriched in yaks. This pattern suggests a potential viral-mediated mechanism that supports the superior capacity of yaks to utilize low-quality forage, consistent with their previously reported digestive efficiency advantages over cattle [23, 59]. Given the low temperatures, hypoxic conditions, and seasonal forage limitations characteristic of the Qinghai-Tibet Plateau, such virus-associated metabolic functions may help sustain rumen ecosystem function under environmental stress. These findings suggest a potential role of the rumen DNA virome in supporting microbial resilience within high-altitude grazing systems.

Viruses encode diverse anti-defense genes to counter prokaryotic defense mechanisms, reflecting the ongoing evolutionary arms race between viruses and their hosts [60]. In both yaks and cattle, we observed a significant reduction in the diversity of viral anti-defense genes during the cold season. This pattern may reflect altered selective pressures under nutrient-limited conditions. During the cold season, microbial communities are likely to allocate resources toward metabolic maintenance rather than costly immune defenses such as CRISPR-Cas and restriction-modification systems, consistent with the energy trade-off hypothesis [61]. In addition, rumen bacterial diversity was previously shown to decline during the cold season in both yaks and cattle [24], potentially reducing the diversity of host defense mechanisms available within the microbial community [62]. Together, these changes may lessen the selective pressure for maintaining a diverse repertoire of viral anti-defense genes. Beyond seasonal trends, several host-specific anti-defense genes, including *vcrx091*, *vcrx093*, and *tad2*, were detected exclusively in cattle, indicating distinct anti-defense gene reservoirs between host species. Notably, cattle exhibited enrichment of the anti-CRISPR gene *vcrx093* and the anti-restriction modification gene *mom* relative to yaks, which may reflect differences in virus-host interaction dynamics and defense-counter processes between the two host species. Collectively, these findings indicate that viral anti-defense systems are shaped by both seasonal environmental conditions and host-specific ecological contexts, further highlighting the dynamic nature of virus-host coevolution within the rumen ecosystem.

Several limitations should be acknowledged in this study. First, although significant seasonal and host-specific differences were observed, the sample size may have limited the ability to fully capture inter-individual variation in the rumen virome. Second, given the largely unexplored nature of rumen viral diversity, a relatively inclusive approach was adopted for vOTU retention to maximize viral recovery. Because genome quality estimates from tools such as CheckV depend on incomplete reference databases for rumen viruses, some genuine viral genomes may be underestimated in quality. Consequently, restricting analyses to only medium- and high-quality vOTUs could result in the loss of substantial viral diversity. Third, integration of bacterial and viral community dynamics was limited by the incomplete assignment of vOTUs to putative microbial hosts, reflecting current limitations in host prediction methods and reference genome databases. Nevertheless, bacterial community profiles from the same cohort have been previously characterized [24], enabling focused analysis of dominant microbial taxa and their associations with key vOTUs across seasons. Finally, the present study was based on DNA metagenomics and therefore did not capture RNA viruses or retrovirus-like elements, representing only a subset of the total rumen virome. In particular, retrovirus-like elements, including integrated viral sequences capable of long-term persistence in host genomes [63], were not resolved in this study. Future studies incorporating larger cohorts, comprehensive viromics (covering both DNA and RNA viruses), and improved virus-host linkage approaches will be required to obtain a more comprehensive understanding of rumen viral ecology.

## Conclusion

In summary, this study demonstrates that the rumen DNA virome of grazing yaks and cattle exhibits pronounced seasonal and host-specific dynamics in response to warm-to-cold environmental transitions on the Qinghai-Tibet Plateau. Seasonal shifts in viral community composition, virus-host associations, AMGs, and anti-defense genes suggest that viruses may contribute to microbial adaptation to nutrient-limited winter conditions. The enrichment of AMGs involved in energy metabolism, fatty acid metabolism, and plant fiber degradation during the cold season highlights the potential role of viruses in supporting microbial metabolic flexibility and ecosystem resilience. Compared with cattle, yaks exhibited greater virome stability and a stronger enrichment of metabolism-related AMGs, highlighting distinct host-specific virome responses to environmental challenges. Collectively, these findings provide new insights into rumen viral ecology in high-altitude grazing systems and suggest that the virome may represent an important component of microbiome-mediated adaptation under seasonal nutritional stress. This study also highlights the potential relevance of rumen DNA virome for developing microbiome-informed strategies to improve ruminant resilience and productivity in extreme environments.

## Supplementary Information


Additional file 1: Table S1. Taxonomic classification of viral operational taxonomic units (vOTUs). Table S2. Predicted lifestyles of vOTUs (virulent or temperate phages). Table S3. Predicted microbial hosts of vOTUs. Table S4. Summary information of vOTUs retained for downstream analysis. Table S5. KEGG functional annotation of representative viral genes. Table S6. CAZyme annotation of representative viral genes. Table S7. Differentially abundant KEGG pathway between yak and cattle across seasons. Table S8. Differentially abundant CAZyme profiles between yak and cattle across seasons. Table S9. Virus-host linkage profiles across seasons and host species.Additional file 2: Fig. S1. Viral diversity of yak and cattle across seasons. A Comparison of viral alpha diversity between yak and cattle within each season. B Beta diversity analysis of viral community similarity between yak and cattle. ^*^P < 0.05, ^**^P < 0.01, ^***^P < 0.001. Fig. S2. Differentially abundant viral families in yak and cattle across cold and warm seasons. Fig. S3. Principal coordinate analysis (PCoA) of virus-encoded KEGG Orthologys (KOs) and CAZyme profiles based on the Bray–Curtis distances. Fig. S4. Top 10 viruses with the highest metabolic gene content. A KO gene counts encoded by the top viruses. B CAZyme gene counts encoded by the top viruses. Top KO-rich viruses belong to the family Peduoviridae, while CAZyme-rich viruses are unclassified Caudoviricetes. Fig. S5. Differentially abundant KEGG pathways between yak and cattle across seasons. Fig. S6. Anti-defense gene profiles in yak and cattle. A Shared and unique anti-defense genes between yak and cattle. B Differentially abundant anti-defense genes between yak and cattle within each season. Venn diagram was generated using Venny 2.1 (https://bioinfogp.cnb.csic.es/tools/venny/).

## Data Availability

The raw data are available from the NCBI Sequence Read Archive (SRA) under the accession number PRJNA734085.

## Abbreviations

AMG: Auxiliary metabolic gene
CAZyme: Carbohydrate-active enzyme
KOs: KEGG Orthologs
ORF: Open reading frame
vOTU: Viral operational taxonomic unit
