# Supplementary material for: Rumen DNA virome plasticity and viral metabolic potential are associated with seasonal adaptation in grazing yak and cattle on the Qinghai-Tibet Plateau
Source: J Anim Sci Biotechnol. 2026 Sep 1;17:171. doi: 10.1186/s40104-026-01487-8 (PMC13531870; doi:10.1186/s40104-026-01487-8)
Supplement: Supplementary file 1 [file 40104_2026_1487_MOESM2_ESM.docx]

**Fig. S1.** Viral diversity of yak and cattle across seasons. **A** Comparison of viral alpha diversity between yak and cattle within each season. **B** Beta diversity analysis of viral community similarity between yak and cattle. ^*^*P* < 0.05, ^**^*P* < 0.01, ^***^*P* < 0.001.

**Fig. S2.** Differentially abundant viral families in yak and cattle across cold and warm seasons.

**Fig. S3.** Principal coordinate analysis (PCoA) of virus-encoded KEGG Orthologys (KOs) and CAZyme profiles based on the Bray–Curtis distances.

**Fig. S4.** Top 10 viruses with the highest metabolic gene content. **A** KO gene counts encoded by the top viruses. **B** CAZyme gene counts encoded by the top viruses. Top KO-rich viruses belong to the family *Peduoviridae*, while CAZyme-rich viruses are unclassified *Caudoviricetes*.

**Fig. S5.** Differentially abundant KEGG pathways between yak and cattle across seasons.

**Fig. S6.** Anti-defense gene profiles in yak and cattle. **A** Shared and unique anti-defense genes between yak and cattle. **B** Differentially abundant anti-defense genes between yak and cattle within each season. Venn diagram was generated using Venny 2.1 (https://bioinfogp.cnb.csic.es/tools/venny/).
